# Association of Zinc Transporter-8 Autoantibody (ZnT8A) with Type 1 Diabetes Mellitus

**DOI:** 10.7759/cureus.7263

**Published:** 2020-03-13

**Authors:** Afreen Bhatty, Saeeda Baig, Asher Fawwad, Zil E Rubab, Moazzam A Shahid, Nazish Waris

**Affiliations:** 1 Biochemistry, Ziauddin University, Karachi, PAK; 2 Medicine, Baqai Institute of Diabetology and Endocrinology, Baqai Medical University, Karachi, PAK; 3 Research, Baqai Institute of Diabetology and Endocrinology, Karachi, PAK

**Keywords:** autoimmune disease, zinc transporter 8, autoantibodies, autoimmunity, diabetes mellitus, type 1

## Abstract

Background: Zinc transporter 8 autoantibody (ZnT8A), discovered through bioinformatics, is identified as another major biomarker for type 1 diabetes mellitus (T1DM), expanding the panel of diagnostic autoantibodies. The absence of standard autoantibodies in T1DM patients and the presence of ZnT8A in individuals before disease development has led the researchers to evaluate ZnT8A to gather information about the frequency and its association. Therefore, we aim to find out the concentration of ZnT8A and its association with T1DM.

Methods: A case-control study with 25 type 1 diabetes mellitus patients and 25 first-degree relatives of cases as controls was conducted at Ziauddin University in collaboration with the Baqai Institute of Diabetology and Endocrinology (BIDE), Karachi. Demographic data were collected from patients on a standard questionnaire. Blood samples were collected, after approval from Ziauddin Ethics Review Committee, from subjects and serum was separated to estimate ZnT8A by using sandwich enzyme-linked immunosorbent assay (ELISA).

Results: The mean age at diagnosis of T1DM patients was 13.40±5.05 years, and the duration of diabetes was 7.74±5.85 years. The frequency of ZnT8A was found higher in cases (19 (76%)) compared to controls (6 (24%)). ZnT8A concentrations were significantly higher in cases (13.82 ng/ml) compared to the controls (8.78 ng/ml; p= 0.024). The cut-off value of 9 ng/ml was selected for measuring sensitivity, specificity, and accuracy, which were determined as 76%, 76%, and 76%, respectively.

Conclusions: ZnT8A was found significantly associated with T1DM. Subjects with ZnT8A values ≥ 9 ng/ml are 10 times more at risk to develop T1DM (p = 0.000).

## Introduction

Type 1 diabetes mellitus (T1DM) is characterized by autoimmune destruction of pancreatic β cells, the targets being autoantigens showing high expression and β-cell specificity [1]. It is estimated to be 5-10% of the total diabetic cases globally and is predicted to surge by 3% per year [2]. Furthermore, it is a chronic disease presented around 5-7 years of age till puberty but can be diagnosed at any age [3]. The prevalence of T1DM in Asian populations is very low (0.4-1.1 cases/year/100,000 individuals) compared to the other populations where siblings are prone to develop the autoantibodies earlier [4]. Individuals with multiple first-degree relatives with T1DM are at higher risk for the development of T1DM [5].

ZnT8 is an autoantigen present mostly in pancreatic β-cells; it is made up of 369 amino acids and is the product of the SLC30A8 gene. The location of this gene is at position 24.11 on the q arm of chromosome 8 [6,7]. ZnT8 is a six-transmembrane protein transporter and a member of the cation diffusion family that facilitates the transport of Zn^+2^ ions from the cytoplasm to insulin vesicles [8,9]. It plays an essential role in the storage, secretion, structural stabilization, and action of insulin [10,11]. Frequent exposure of ZnT8 antigen occurs during the exocytosis of insulin that is stimulated by glucose. ZnT8 exposure in genetically predisposed individuals can aggravate or activate the production of autoantibodies against ZnT8 antigens [8].

Besides genetics, the development of type 1 diabetes mellitus is also attributed to SLC30A8 polymorphism, overexpression of ZnT8, viral infections (Coxsackie, rhinovirus, and adenovirus) and ZnT8 exposure during insulin release [6]. Pathogenic T-cells mediate the autoimmune destruction of pancreatic β-cells by targeting well known β-cell autoantigens [8]. The estimation of autoantibody for diagnostic purposes started a few years ago. Apart from diagnosis, autoantibody determination can be advantageous in evaluating the immunological influence on therapeutic strategies targeting autoreactive B- and T-cells [6].

Autoantibodies against the earlier recognized antigens, like glutamic acid decarboxylase (GAD), insulin, islet antigen 2 (IA-2), were considered standards. Recently recognized as an additional biomarker for the diagnosis of T1DM, ZnT8 was identified through bioinformatics based on the common features including, enhanced β-cell expression, alternative splicing, and association with the secretory pathway. Antibodies against ZnT8 are considered as an independent demonstrator of autoimmunity for the diagnosis of T1DM [8,11,12].

Approximately 26% of T1DM subjects were found positive for ZnT8A, who were negative for antibodies against GAD, IA-2, and insulin antigen [13]. In the Caucasoid population, it has been reported that ZnT8A were discovered in more than 60% of type 1 diabetics, whereas, combined measurement of standard autoantibodies with ZnT8A increased the detection rates to 98% [14]. The absence of standard autoantibodies in T1DM patients and the appearance of ZnT8A in the individuals before the disease development has led researchers to measure and associate these antibodies with T1DM. ZnT8A can add information about the frequency, concentrations, and association with T1DM in our population. Therefore, we aimed to find out the association of ZnT8A with T1DM.

## Materials and methods

This prospective study was conducted at Ziauddin University hospital with the collaboration of Baqai Institute of Diabetology and Endocrinology (BIDE) - Baqai Medical University (BMU). Fifty subjects were recruited for the study, who visited the out-patient department of BIDE-BMU. The duration of recruitment was between June 2019 and Oct 2019 (5-month duration). Subjects were categorized into case and control groups. The case group included T1DM patients (n=25) meeting the revised criteria of the American Diabetes Association (ADA). The first-degree relatives of the cases were recruited as the healthy control group (n=25) with no autoimmune disease.

Serum samples were taken and stored at -80°C. The concentration ZnT8A was measured using a sandwich enzyme-linked immunosorbent assay (ELISA) kit (AEE490Hu, Cloud-Clone Corp., Houston, USA). All the reagents, samples and standards were prepared, 100µL standard or sample was added to each well and incubated for one hour at 37°C. 100µL of prepared detection reagent A was aspirated and incubated for one hour at 37°C. It was then aspirated and washed five times. 90µL of the substrate solution was added and incubated for 10-20 minutes at 37°C. Stop solution (50µL) was added at the end to terminate the reaction. The color change was measured spectrophotometrically at 450nm. The concentrations were then analyzed by comparing the optical density of the samples with the standard curve.

Statistical analysis was carried out using the Statistical Package for the Social Sciences (SPSS) 20.0. The results of numeric variables were expressed as mean and standard deviation for parametric data and median and range for non-parametric data. Categorical data were expressed as frequencies and percentages. A p-value <0.05 was considered statistically significant. Chi-squared and Fisher’s exact tests were employed to compare the differences in the distribution of categorical variables (gender and ethnicity) between cases and controls. Independent t-test and Pearson’s correlation were used to compare the differences in the concentration of autoantibodies with age, gender, and ethnicity.

## Results

Out of 25 cases, 14 (56%) were males and 11 (44%) females with the mean age of 20.96±6.34 years. In the control group, 10 (40%) were males and 15 (60%) females with the mean age of 42.12±11.20 years. The mean age at diagnosis of T1DM patients was 13.40±5.05 years, and diabetes duration was 7.74±5.85 years. The demographic data of T1DM patients are summarized in Table 1.

**Table 1:** General Characteristics of Patients with Type 1 Diabetes Mellitus and Controls (A) Data are presented as the number of subjects and percentages. (B) Data are presented as mean and standard deviation. p-value <0.05 is considered statistically significant. * p-value <0.0.5

A		CASES n (%)	CONTROLS n (%)	p-VALUE
Gender	Male	14 (56%)	10 (40%)	0.406
	Female	11 (44%)	15 (60%)	
Ethnicity	Urdu speaking	12 (48%)	12 (48%)	0.147
	Sindhi	4 (16%)	4 (16%)	
	Punjabi	2 (8%)	2 (8%)	
	Balochi	4 (16%)	4 (16%)	
	Others	1 (4%)	1 (4%)	
Educational level	Illiterate	3 (12%)	9 (36%)	0.025*
	Primary/Secondary	14 (56%)	9 (36%)	
	Intermediate	6 (24%)	3 (12%)	
	Graduate	2 (8%)	4 (16%)	
B		CASES (Mean ± SD)	CONTROLS (Mean ± SD)	p-VALUE
Age (years)		20.96±6.34	42.12±11.20	0.000*
Disease duration (years)		7.74±5.85	-	
Age at diagnosis (years)		13.40±5.05	-	
Height (cm)		159.29±7.26	158.08±22.94	0.819
Weight (kg)		55.00±10.70	69.45±16.17	0.001*
Systolic BP (mm/Hg)		106.80±13.54	117.50±13.71	0.012*
Diastolic BP (mm/Hg)		72.6±12.00	82.05±10.70	0.009*

**Figure 1:**
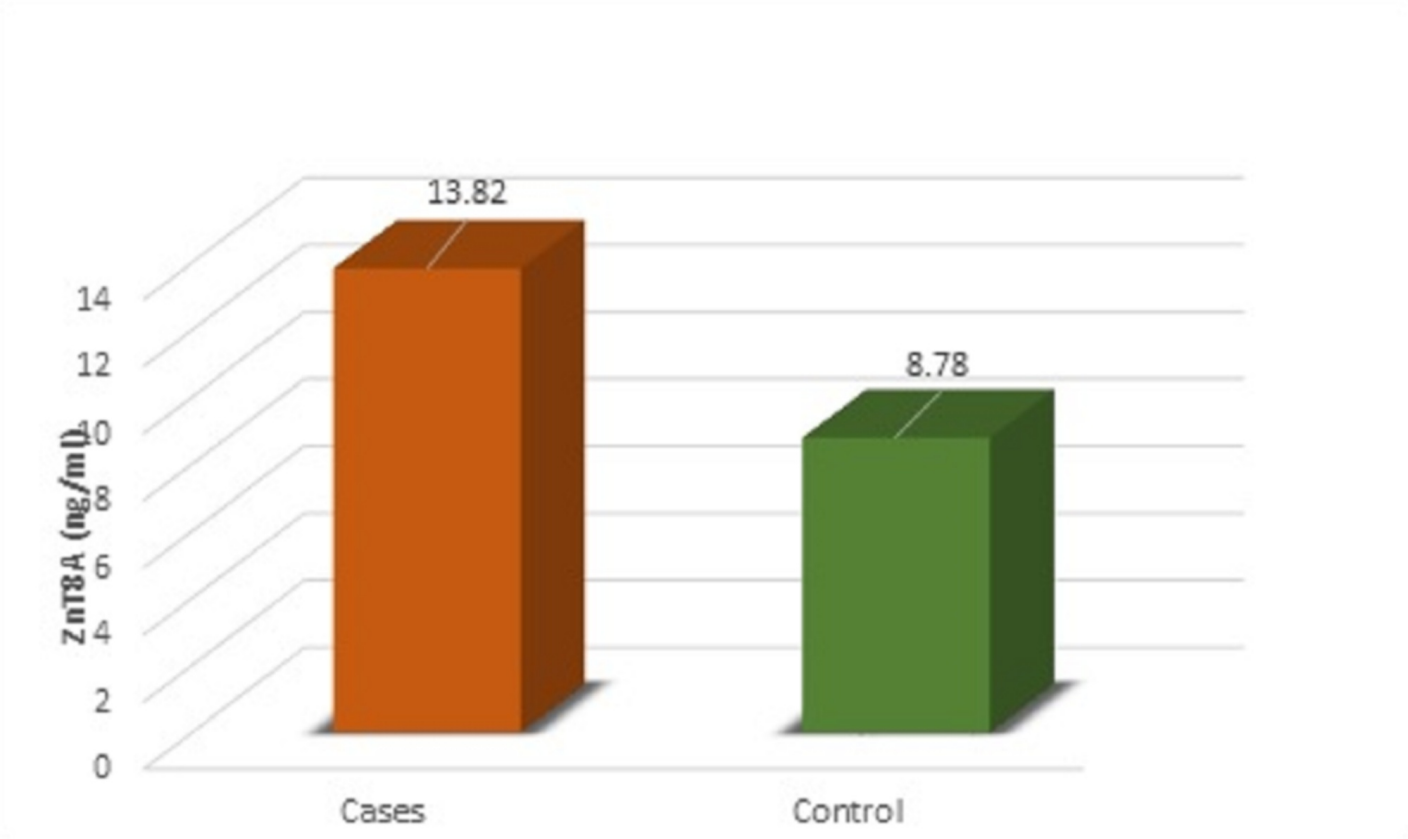
Mean Concentration of ZnT8A Showing Significant Differences Between Cases and Controls

Among the cases, 60% were less than eight years of age, and 88% were dependent on insulin. The mean concentration of ZnT8A in the case group is 13.82 ng/ml, while in the control group, the mean is 8.78 ng/ml as showing significant differences in Figure 1. ZnT8A was identified in 19 (76%) cases with a cut-off value of 9 ng/ml.

The analysis revealed that the concentration of ZnT8A in cases with positive ZnT8A were significantly higher compared to the levels of ZnT8A+ in the control group (p = 0.024). ZnT8A were found similar between males and females. The ZnT8A levels were not associated with age, gender, and ethnicity.

The ZnT8A concentration between the males and females were analogous (12.29 ng/ml vs. 10.39 ng/ml; p=0.406). There was no difference between Urdu speaking, Punjabi, Pathan, Balochi, and other ethnicities (p=0.442). Among the 25 healthy first-degree relatives of T1DM patients, six (24%) were positive for ZnT8A, one of them showed high levels of 33.32 ng/ml. The cut-off value of 9 ng/ml was tested for the sensitivity, specificity, and accuracy and was found 76%, 76%, and 76%, respectively.

## Discussion

In this study, we found that the ZnT8A concentrations in the T1DM group were significantly higher (13.82±7.92 ng/ml) than in controls (8.78±7.33 ng/ml). Among the controls, six (24%) were positive for ZnT8A; one of them showed a high level at 33.32 ng/ml. A higher concentration of ZnT8A+ in six subjects from the control group raises the possibility of them developing T1DM later in life. ZnT8A was found significantly associated with T1DM (p = 0.000). A Brazilian study reported similar results [7]. Controls with a higher concentration of ZnT8A are 10 times more at risk for the development of T1DM.

The majority of the research done in this field was in the admixture population like Asians, Caucasians, and Brazilian; their results remain uncertain [7,14-18]. There was no definite conclusion from the above-mentioned studies; therefore, this study can add information about the relationship between ZnT8A and T1DM.

The cases and the controls in the current study belong to the Asian ancestry. The age at diagnosis of patients with type 1 diabetes was 13.40±5.05 years, and the duration of diabetes was 7.74±5.85 years. The mean age at the onset of diabetes was similar to an Indian population, and the distribution of gender was also similar [8]. There was a higher frequency of ZnT8A in males in the current study, which contrasts with a number of studies that reported a higher frequency in women or even documented no difference between genders with T1DM that was noticed by Eisenbarth et al. probably because of difference in gender distribution in these studies [15,19-21].

In the current study, the frequency of ZnT8A was found higher (76%) in diagnosed cases of T1DM. This data is similar to the incidence in Caucasian and Brazilian populations but higher than those in a study with Asian ancestry probably due to a fewer number of Asians is unable to depict the exact representation of the population [7,16,17,22]. ZnT8A was found positive in six (24%) individuals in the control group. This result is in accordance with most of the studies done in other countries [8,14,23].

In a program of diabetes antibody standardization, the sensitivity of ZnT8A was predicted as 55%, while the specificity as 98% [14]. The cut-off value of 9 ng/ml was taken and tested in the current study for the sensitivity, specificity, and accuracy, which came out 76%, 76%, and 76%, respectively. The ranking order of ZnT8A was found better than insulin autoantibodies (IAA) among the islet antibodies in a Caucasian population, increasing the diagnostic sensitivity by 2% [15]. The limitations of our study were smaller sample size and lack of other antibodies' (insulin antibody (IA), glutamate decarboxylase antibody (GADA), and tyrosine phosphatase-related islet antibody (IA-2A)) measurement.

## Conclusions

ZnT8A were higher in T1DM cases than in controls. However, similar results were found between genders and among different ethnicities. ZnT8A were significantly associated with T1DM. Controls with a positive family history of T1DM and ZnT8A values ≥ 9 ng/ml are 10 times more at risk of developing T1DM. More studies should be done to make it a diagnostic biomarker and to test the sensitivity, specificity, and accuracy of autoantibodies at different cutoff values. Individuals with a positive family history of T1DM should be screened for these autoantibodies for early diagnosis.
